# Constitutive metanephric mesenchyme-specific expression of interferon-gamma causes renal dysplasia by regulating Sall1 expression

**DOI:** 10.1371/journal.pone.0197356

**Published:** 2018-05-17

**Authors:** Kangsun Yun, Arthur A. Hurwitz, Alan O. Perantoni

**Affiliations:** 1 National Cancer Institute/NIH, Cancer and Developmental Biology Laboratory, Frederick, MD, United States of America; 2 National Cancer Institute/NIH, Laboratory of Molecular Immunoregulation, Frederick, MD, United States of America; UCL Institute of Child Health, UNITED KINGDOM

## Abstract

Transplacental viral and parasitic infections have been shown to initiate an innate response in the mammalian embryo by increasing the expression of pro-inflammatory cytokines such as interferon-gamma (Ifng). However, the developmental consequences of an activated innate immunity and, in particular, the effects of induction of Ifng expression independent of infection have been largely overlooked. Here, we demonstrate *in vivo* that the conditional overexpression of Ifng in metanephric mesenchymal (MM) progenitors results in renal agenesis or hypoplasia. Cell death was observed in and around the MM region of E10.5–11.5 mutants where Ifng was constitutively expressed during early kidney development and resulted in a retardation of branching morphogenesis. Furthermore, isolated mutant or normal Ifng-treated metanephroi replicated this phenotype in culture, demonstrating the inherent nature of the aberrant morphogenesis. The expression of renal progenitor marker Sall1 was significantly decreased in the MM of mutant kidneys, suggesting that a reduction in *Sall1* may be the cause of cell death in the MM during early kidney development and that, in turn, retards UB branching in the mutants. Therefore, the aberrant induction of Ifng expression, as part of an innate immune response, may contribute to renal agenesis or hypoplasia during early metanephric development by regulating the MM progenitor population.

## Introduction

Among the most common birth defects in humans are congenital anomalies of the kidney and urinary tract (CAKUT). Kidney dysplasia is one form of CAKUT and can be detected by pre- or postnatal ultrasound as an abnormally sized kidney. Unilateral renal dysplasia occurs in 1 in 1000 births and the bilateral form, which can lead to severe kidney dysfunction and mortality, in 1 in 5000 births. Kidney dysplasia is usually caused by deficient branching morphogenesis and/or abnormal nephrogenesis [1]. The kidneys arise from the intermediate mesoderm, and their development is mediated by reciprocal signaling interactions between the metanephric mesenchyme (MM) and ureteric bud (UB). Renal agenesis results from defects in genes that regulate initial UB outgrowth such as *Gdnf*, *Ret*, and *Gfra1*. Also, the loss of certain genes, for example *Eya1*, *Six1*, *Hox11*, *Wnt4*, and *Fgf8*, which are involved later during metanephric development or nephron patterning, causes renal agenesis or hypoplasia [2]. *Sall1* knockout (KO) mice are subject to renal malformation as well. Sall1 regulates *Kif26b* [3,4] and branching initiation in the metanephros by modulating Wnt9b signaling at the UB tip [5]. Since it participates in the maintenance of multipotent renal progenitors, Sall1 is essential for tissue regeneration [6].

In addition to genetic defects, extrinsic factors to which the embryo/fetus are exposed in utero, such as teratogens/drugs [7–11], maternal diet [12,13], hypoxia [14], or even inflammation [15–18] may also affect kidney development [1,19,20]. A reduced nephron number is an indicator of renal hypoplasia and correlates with low birth weight following intrauterine insults [20]. These reductions in nephrons can lead to chronic conditions later in life, such as hypertension or hyperfiltration [21], so understanding the factors that regulate nephron numbers may have significant biological ramifications. However, to date the role of various extrinsic factors in renal development has not been investigated.

Transplacental infections such as those initiated by cytomegalovirus can induce fetal inflammatory response syndrome, triggering organ dysplasia and brain injury [17,22]. Embryonic infection by Zika virus restricts intrauterine growth and causes microcephaly in mice due to an increase in neural progenitor death [23]. For these types of agents, inflammation mediated by innate immunity may be a component of neurodegeneration in response to viral infection in mice. Ifng is a major pro-inflammatory cytokine that is often greatly elevated during inflammation [24]. It functions as a pleiotropic cytokine critical to host defenses in combating viral infections, but it is also complicit in some pathological conditions, such as multiple sclerosis, autoimmune arthritis, and insulin-dependent diabetes mellitus [25]. Ifng is also involved in the maintenance and regeneration of tissues by modulating progenitor populations [26–29]. It regulates embryonic hematopoietic stem and progenitor cell production [30] and can affect organ development in such tissues as the eye and brain [31,32]. For example, constitutive *Ifng* expression in the lens alters the developmental fate of future fiber cells, resulting in abnormal eye development [31]. Furthermore, when targeted to astrocytes, it induces hypomyelination, causing neural dysplasia [25]. All these reports raise the possibility that increased Ifng expression following embryonic infection may contribute to abnormal embryonic organ development. Indeed, several embryonic tissues are likely competent to respond to Ifng, as they produce cognate receptors, *Ifngr1 and Ifngr2* (www.emouseatlas.org). Furthermore, these receptors are demonstrable in early embryos [33] as well as in developing organs, including the kidney (www.informatics.jax.org/marker/MGI:107654).

We have previously shown that Ifng inhibits the differentiation of renal progenitors in MM explant cultures [34]. Here, we further investigate the effects of *Ifng in vivo* by inducing ectopic expression in the MM progenitor. Kidney development was grossly abnormal in the *Ifng* gain-of-function (GOF) mouse, yielding hypoplasia or complete renal agenesis. Cell death was observed in and around the MM region of mutants where *Ifng* was ectopically expressed and was concomitant with a retardation of UB branching morphogenesis. Similarly, branching morphogenesis was dramatically reduced in isolated metanephric explants treated with recombinant Ifng. This morphogenetic outcome is reminiscent of the *Sall1* KO kidney, which is also characterized by MM cell death and retarded UB branching [3]. Moreover, the expression of nephron progenitor marker Sall1 was decreased in *Ifng* GOF mutant kidneys. Therefore, aberrant increased *Ifng* expression in the developing embryo, as part of an innate immune response possibly initiated by a transplacental infection, may contribute to renal agenesis or hypoplasia by regulating the renal progenitor pool through Sall1 modulation. Thus, the *Ifng* GOF mouse may provide a useful model for understanding the pathological effects mediated by innate immunity during embryonic development.

## Materials and methods

### Animals and tissue culture

**Fig 1:**
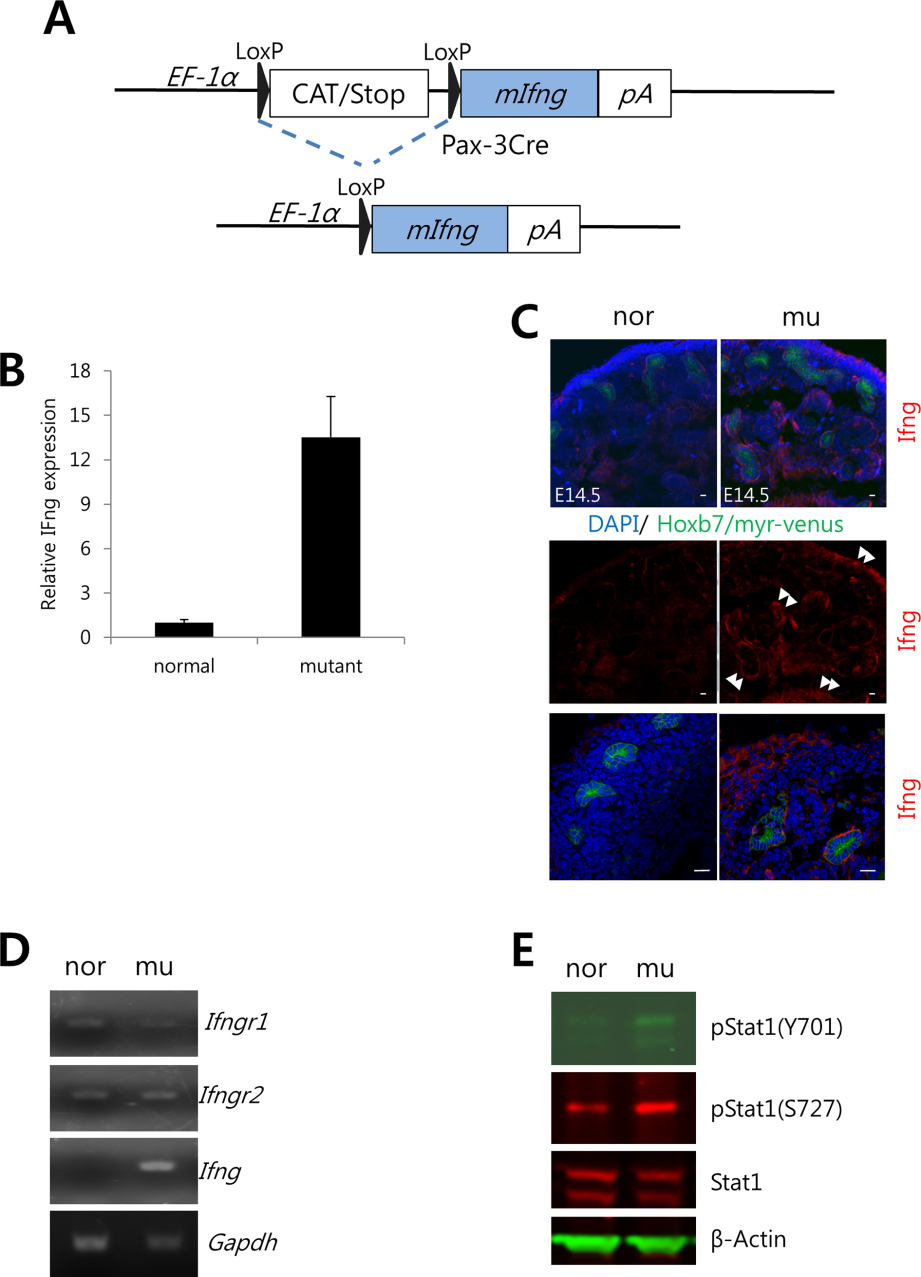
Expression of Ifng increases in the kidneys of the *Ifng* GOF mouse. A. Schematic depicting the Ifng GOF mouse line. Murine Ifng is ectopically expressed in the Pax3-expressing domain after Cre-mediated excision of a LoxP-CAT-Stop cassette. B. The amount of Ifng in kidneys increases about 13 fold in mutant vs. normal kidneys, when *Ifng* expression is targeted to the MM. The amount of Ifng was measured by ELISA. C. Ifng expression (red) is elevated in the MM-derived areas. The UB is marked with Hoxb7/myr-venus (green). Scale bar = 20 μm. D. *Ifng* mRNA is elevated in kidneys from *Ifng* GOF mice. mRNA is measured by semi-quantitative RT-PCR. *Ifngr1* and *Ifngr2* are also detected in mouse embryonic kidneys. E. Stat1 protein is activated in mutant kidneys, as determined by phosphorylation of Y701 and S727 in immunoblots. As a loading control, β-Actin was used. All these experiments used E14.5 kidneys. nor—normal, mu—mutant.

Mice were managed under ASP protocol #16–211, which was approved by the NCI-Frederick ACUC, according to NIH guidelines for the care and use of laboratory animals. Pregnant females were euthanized with CO2 from a gas cylinder. The *Ifng* GOF mouse (C57BL6) was generated by cloning murine *Ifng* cDNA into a LoxP-flanked chloramphenicol (CAT)-polyA stop sequence cassette (LoxPCatstop) to induce *Ifng* expression in a tissue-specific manner (Fig 1A).[35] Generation, maintenance and genotyping of *Ifng* GOF mice, *Pax-3 Cre* mice, [36] and *Hoxb7-myr/Venus* reporter mice [37] were described previously [38]. Noon on the day of vaginal plug detection was considered as E0.5. Mice were managed under an approved protocol, according to NIH guidelines for the care and use of laboratory animals. Metanephric rudiments were dissected from E11.5 mouse embryos and were cultured on type IV collagen-coated filters, as previously described [38], in DMEM/F12(50:50) with 10% fetal calf serum in 7% CO2 at 37°C.

### Whole-mount in situ hybridization (WISH) and immunostaining

Embryos were fixed and processed for WISH as described [38]. Also, isolated metanephroi and cultured tissues were processed as described for immunostaining [38]. Calbindin staining of kidney explant cultures was performed with calbindin polyclonal antibody (Millipore) according to a protocol for immunostaining as described [38]. Sall1 staining of kidney transverse sections was performed with a 1:100 dilution of Sall1 antibody (Abcam) and Alexafluor 568 secondary antibodies (Invitrogen) at 1:2,000 dilution. Immunostaining with Ifng (BioLegend) and IRF6 (Aviva Systems Biology) was performed with a 1:50 antibody dilution and Tyramide Signal Amplification (Invitrogen) according to the manufacturer’s instructions.

### Proliferation and cell death studies

Proliferation was analyzed by examining histone H3 phosphorylation using an antibody against the ser10 phosphopeptide of histone H3 (Millipore). Cell death was examined with an *In Situ* Cell Death Detection kit (Roche) according to the manufacturer’s instructions.

### Semi-quantitative RT-PCR

Total RNA was purified using TRIzol reagent (Invitrogen), and reverse-transcribed with a Superscript III RT kit (Invitrogen) according to manufacturer's instructions. PCR reactions were performed using a PCR enzyme master mix (Roche) with primers. PCR conditions were 94°C for 30 s, 60°C for 30 s and 72°C for 30 s. PCR products were resolved in 1.5% agarose gels. Primer sequences, annealing temperatures, and cycle parameters are provided in S1 Table.

### Immunoblotting and ELISA

For immunoblotting, kidney lysates were prepared as previously described [39]. Proteins were detected with specific antibodies against Stat1 (Cell Signaling), phospho-Stat1 (Cell Signaling), β-Actin (Sigma) and active β-Catenin (Millipore). Blots were developed using a peroxidase-conjugated secondary antibody (Sigma) and an enhanced SuperSignal West Pico Chemiluminescent Substrate system (ThermoFisher Scientific) according to manufacturer's instructions. For ELISA, lysates were prepared from kidneys at E14.5. The amount of the Ifng was measured with a mouse Ifng Femto-High Sensitivity ELISA kit (eBioscience).

## Results

### Expression of Ifng is increased in the kidneys of Ifng GOF mice

In the embryonic kidney, *Ifng* is normally expressed weakly in isolated MMs (demonstrable after 40 PCR cycles), whereas it is not detectable in the UB. *Ifngr1 and Ifngr2* were detected in both the MM and UB after only 33 cycles, suggesting that cells in both populations of the metanephros are competent to respond to Ifng and that the ligand can therefore induce signaling to activate downstream targets during development (S1 Fig). Progenitor-localized expression of MM markers, *Six2* and *Gdnf*, and UB marker *c-Ret* confirmed the quality of the tissue separations.

To target and induce *Ifng* levels in the MM, we used a transgenic line bearing an insertion composed of a LoxP/CatStop cassette upstream of the mouse *Ifng* sequence (S2A Fig). Cre activation results in the removal of the stop codon, allowing the tissue-targeted induction of *Ifng* expression. For this, we used a mouse line with *Pax-3 Cre*, which, in the kidney, is active in the MM but not in the UB [36]. Cre-mediated recombination is robust in the nephrogenic cords, including the point of UB outgrowth at the initiation of metanephric development, in E10.5 embryos [40]. Under these conditions, Ifng protein levels were increased about 13 fold in E14.5 embryos (Fig 1B). Elevated expression of Ifng was observed in and around most MM progenitor-derived populations, including cortical and interstitial stroma and nephronic epithelia, and lines receptor-bearing UB epithelia (Hoxb7/myr-venus reporter) in mutant kidneys (Fig 1C). This pattern is consistent with Pax3-Cre recombination in the kidney [36]. To identify those cells that are responding to Ifng, we analyzed the expression of Interferon Regulatory Factor 6 (IRF6), a marker of adult renal epithelia, having observed an increase in IRF6 expression by microarray analysis of MMs from GOF mutants at E11.5 (personal observation). IRF6, which is weakly expressed in normal renal stroma and renal epithelia, is increased in both nephronic epithelia (white arrowheads) and the UB (Hoxb7/myr-venus reporter; white arrows) in GOF mutants (S2B Fig), suggesting that the induction of Ifng in the MM is affecting the UB as well as the MM. This pattern corresponds with the observed expression of *Ifng receptors* by both progenitor populations. We confirmed the increased expression of *Ifng* in mutants by semi-quantitative RT-PCR (Fig 1D). Since Ifng is known to activate JAK/Stat signaling by inducing Stat1 phosphorylation, we also analyzed normal and mutant tissue lysates for pStat1(Y701) and (S727). In the mutant kidneys, we observed elevated levels of both forms of pStat1, consistent with increased *Ifng* expression and activation of the JAK/Stat pathway, with no increase in total Stat1 expression (Fig 1E). These data demonstrate that Ifng signaling is activated in the kidneys of mutant mice.

### Ifng GOF mutants develop renal agenesis or hypoplasia

**Fig 2:**
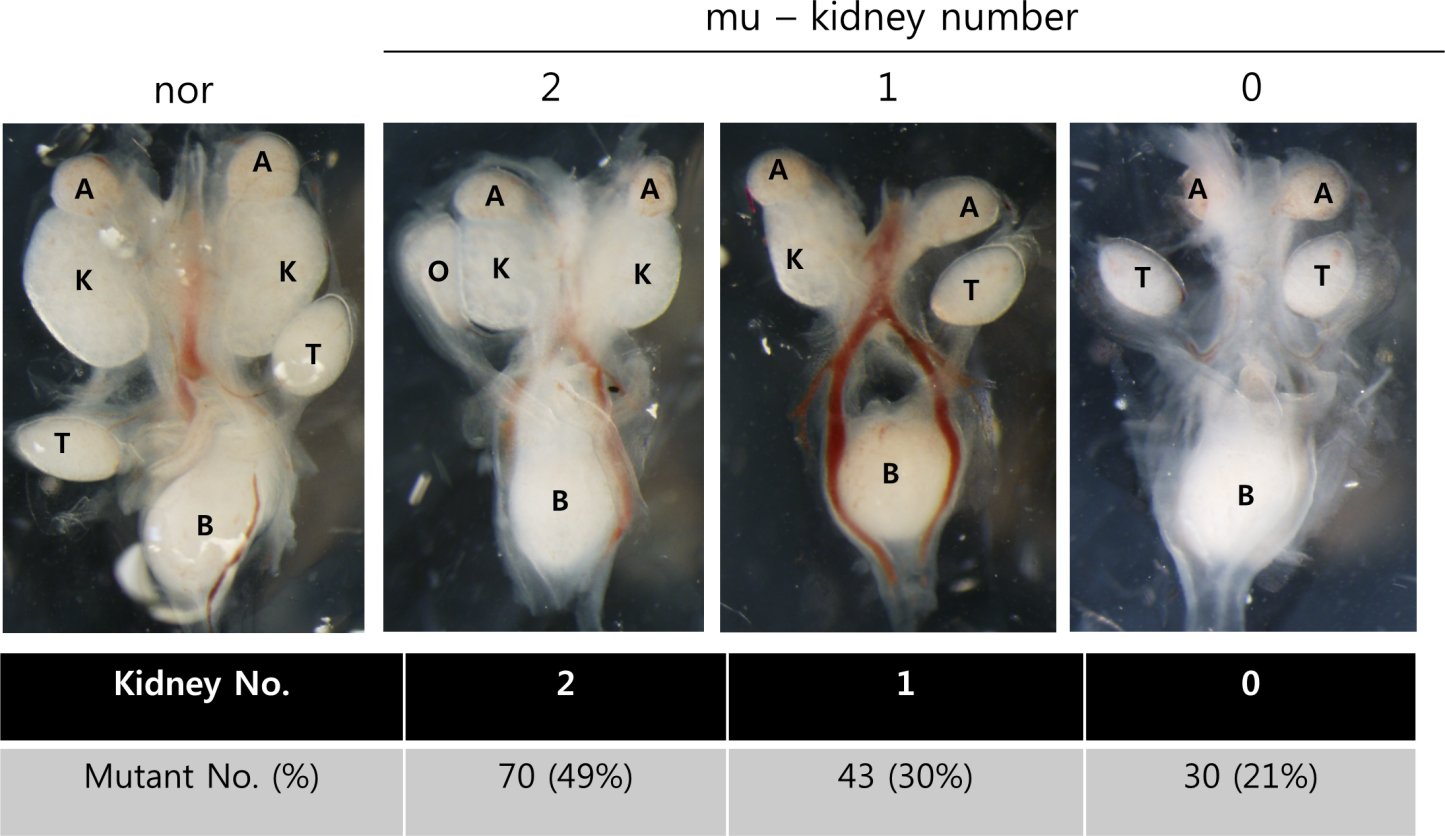
Overexpression of *Ifng* in MM progenitor cells results in renal agenesis or hypoplasia. 49% of mutant embryos have two small kidneys (mu-left), 30% of mutant embryos have one small kidney (mu-middle), and 21% of mutant embryos have no kidneys at E14.5 (mu-right). nor—normal, mu—mutant.

**Fig 3:**
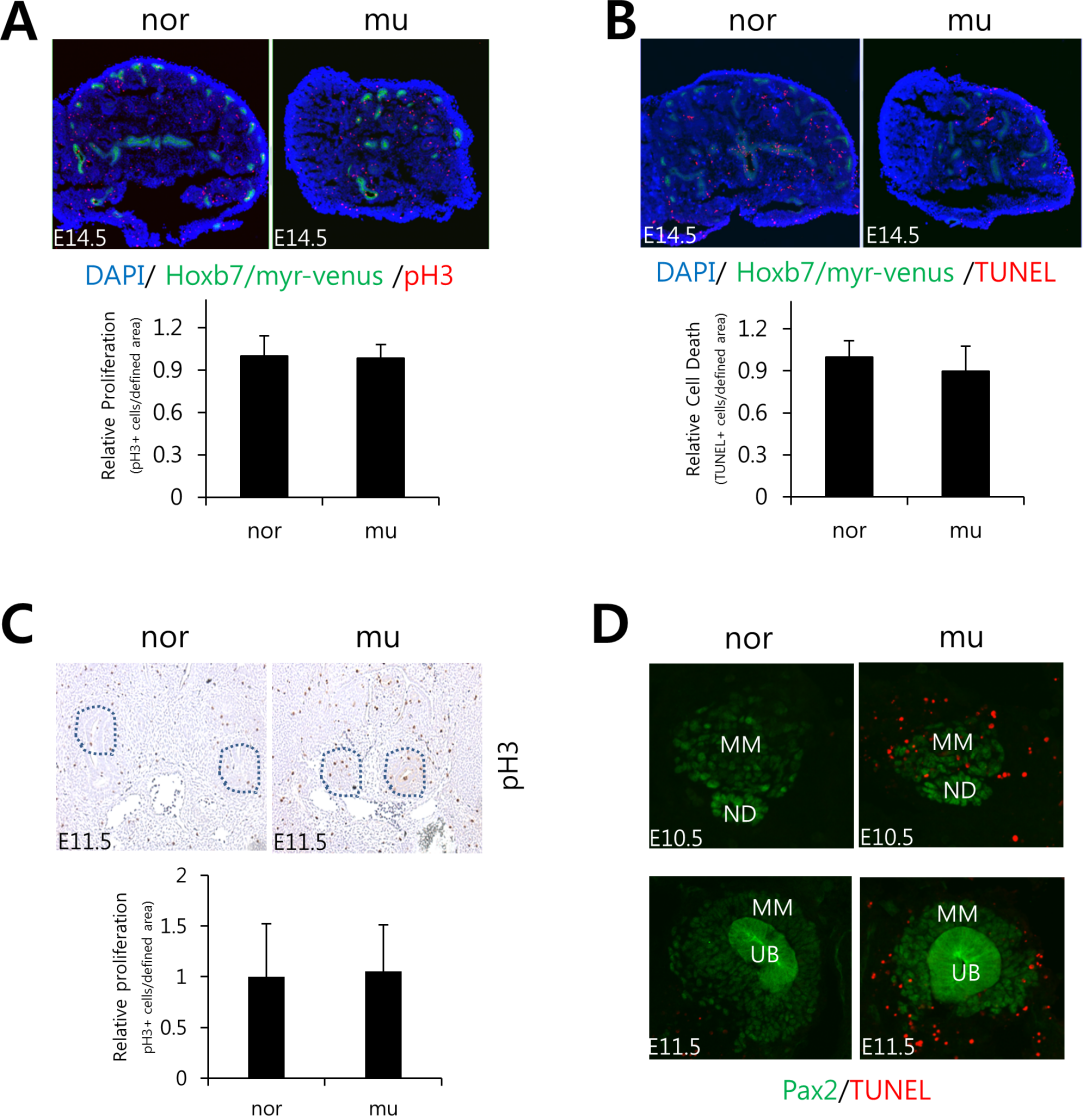
Proliferation and cell death during embryonic kidney development. A. Proliferating cells were detected with phosphohistone 3 antibody in kidneys at E14.5. Proliferation rates over comparable areas are similar between normal and mutant kidneys. B. TUNEL analysis of kidney at E14.5. The incidence of TUNEL-+ cells at this stage over comparable areas is similar between normal and mutant kidneys. C. immunostaining for anti-phosphohistone 3 in proliferating cells in kidneys at E11.5. The dotted line encircles comparable portions of metanephroi. D. TUNEL analysis of kidney at E10.5 (upper) and E11.5 (lower). The kidney is stained with a rabbit anti-Pax2 antibody to demarcate the MM/UB area. The epithelial structure is the nephric duct (ND), and the adjacent Pax2+ tissue delineates the MM region. The UB is also marked with Hoxb7/myr-venus (A, B, D). TUNEL+ cells are numerous in mutant MMs. nor—normal, mu—mutant.

To assess the effect of overexpressed *Ifng*, we analyzed renal development in embryos at E14.5. In GOF mutants, the increased *Ifng* in MM caused congenital renal defects, including renal agenesis or renal hypoplasia. In the latter condition, kidney size was reduced around 30% (Fig 2). Seventy embryos (49%) of 143 total exhibited smaller kidneys bilaterally compared with normal tissues. Thirty percent contained only one kidney, and twenty-one percent showed bilateral agenesis. Of those mutants with kidneys, the organs developed functional nephrons that were properly interfaced with the arborized collecting duct and differed primarily in size. Thus, the animals survived birth, but no effort was made to assess longevity. To determine whether the kidney size difference in mutants was dependent upon a reduction in cell proliferation, we analyzed normal and mutant kidneys for phosphohistone3 (pH3) in tissue sections. Using this approach, we determined that mutant kidneys contained comparable levels of proliferation relative to normal tissues at E14.5 (Fig 3A) and at E11.5 (Fig 3C). Proliferation occurred throughout the developing rudiment in both normal and mutant metanephroi. We then evaluated mutant kidneys for cell death at various stages of development. Interestingly, cell death, as measured by TUNEL staining in sections of E14.5 metanephroi, occurred at comparable levels throughout both the normal and mutant kidneys and was found in the stroma and epithelia, including the collecting ducts (Fig 3B). However, levels of cell death were dramatically increased in and around the MM of E10.5 and E11.5 rudiments (Fig 3D). Pax2 was used as a marker for MM and UB cells in the early rudiments. This result is consistent with the observed renal agenesis or dysplasia in mutants and suggestive of a role for Ifng in regulating the early MM progenitor population. Interestingly, based upon an apparent lack of color overlap in the MM, cell death appeared to occur predominantly in Pax2-negative cells but localized to both the Pax2-positive MM area and the surrounding cells. The MM consists of both Pax2+ (nephron progenitors) and Pax2- (stromal progenitors) [41]. The cells targeted by Ifng therefore may be localized not only to the Pax2-positive MM but also include the area immediately adjacent to the Pax2+positive MM, possibly renal cortical stroma, although renal stromal markers typically do not express at E10.5 [41]. Alternatively, Pax2 expression may be lost prior to cell conversion to TUNEL-positive status, thus precluding co-localization.

### Ifng GOF mutants exhibited reduced UB branching morphogenesis

**Fig 4:**
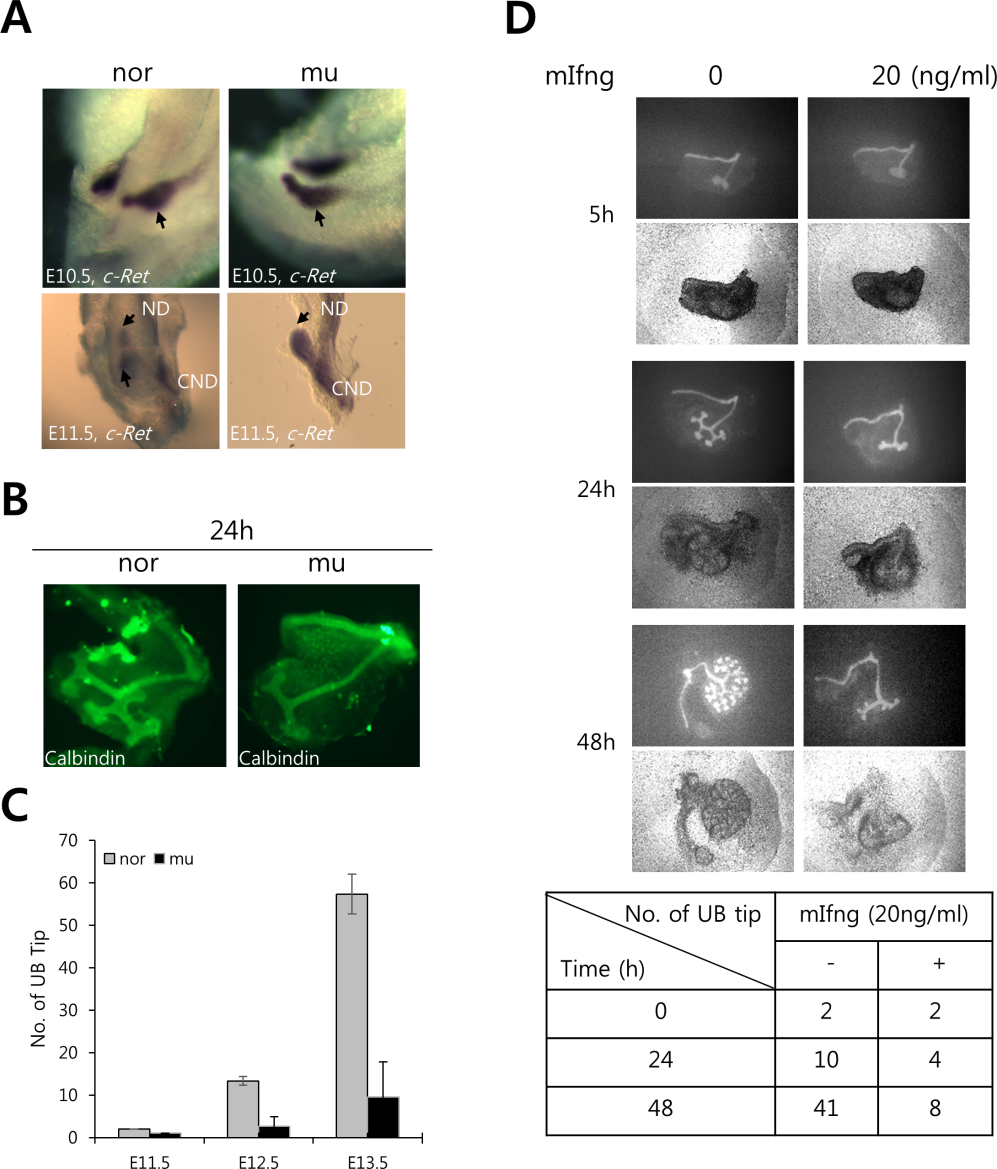
UB branching morphogenesis is retarded in Ifng GOF mutants. A. WISH for *c-Ret* at E10.5 (upper) or at E11.5 (lower). UB tips are marked with black arrows. ND—nephric duct, CND—common nephric duct. B. Calbindin staining of kidney explants cultured for 24h. C. UB tip numbers are significantly reduced in mutants relative to normal embryos during development. Hoxb7/myr-venus containing kidneys were isolated at E11.5, E12.5 and E13.5 and UB tips were counted. D. Hoxb7/myr-venus-expressing cultured kidney explants after treatment with mouse recombinant Ifng (20ng/ml). The fluorescent (upper) or bright field (lower) images were taken from cultures at 5h, 24h and 48h. For explant cultures, E11.5 kidneys were used (B & D). nor—normal, mu—mutant.

Since kidney size is related to the extent of UB branching [42], we examined developing metanephric rudiments for the UB marker *c-Ret*, which is essential for UB morphogenesis. By whole-mount in situ hybridization (WISH), the *c-Ret* expression pattern in mutant and normal rudiments appeared to be comparable at E10.5, showing normal initiation of bud outgrowth from the nephric duct (Fig 4A). However, branching of the UB into the MM was significantly retarded by E11.5 in mutant kidneys. At this stage, the normal embryo had formed a secondary/T-shaped UB branch; whereas, the mutant kidney remained as a primary unbranched outgrowth (Fig 4A). As already mentioned, cell death is elevated in mutant MMs during these early stages, which likely accounts for the retardation in UB branching morphogenesis. We further examined the branching phenotype using explant cultures of isolated metanephric rudiments at E11.5. Over 24 hrs, the normal kidney formed multiple tertiary branches as typically observed *in situ* in the E12.5 kidney, while the mutant kidneys progressed only to the first tertiary branch (Fig 4B). The UB tip numbers were dramatically reduced in mutants and reflect the subsequent renal hypoplasia or agenesis observed in mutants at birth (Fig 4C). This indicates that the observed phenotype is intrinsic to the metanephric rudiment and, therefore, unlikely to be influenced significantly by surrounding tissues. We also examined branching morphogenesis in normal cultured kidney explants with Ifng treatment. In this case, Ifng decreased the rate and extent of branching. In the untreated kidney explants after 48 hrs in culture, we observed branching typical of E13.5 kidneys *in situ*, while the Ifng-treated kidneys remained at the initial stages of tertiary branching (Fig 4D). These data demonstrate that increased expression of or exposure to *Ifng* specifically in the metanephros retards UB branching and again supports the idea that the effects of Ifng are inherent within the metanephros.

### The expression of Sall1 is decreased in Ifng GOF mutant kidneys

**Fig 5:**
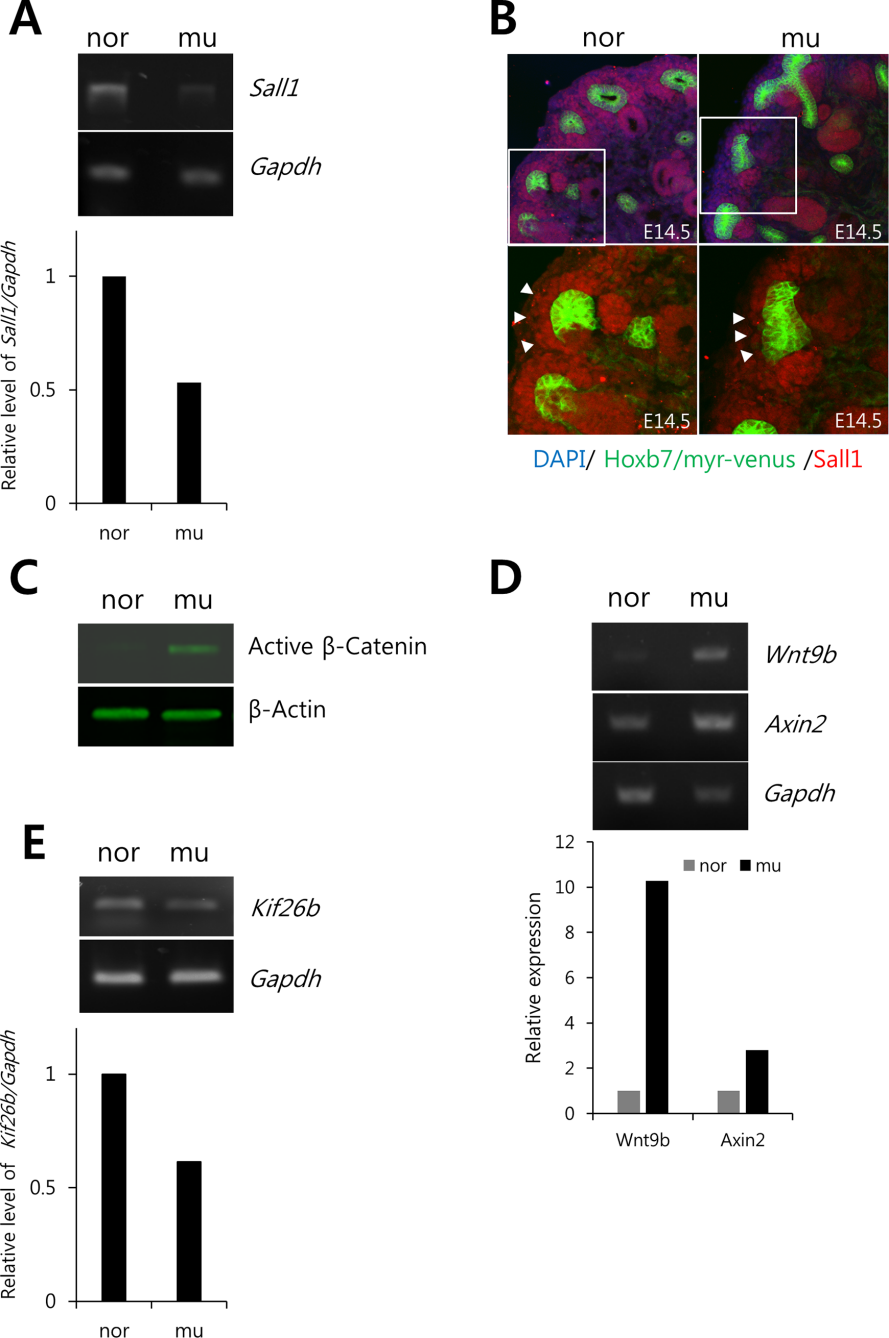
The expression of Sall1 and its potential targets are modulated in mutant kidneys. E14.5 kidneys were used for all analyses. A. semi-quantitative RT-PCR for *Sall1*. The expression level of *Sall1* mRNA is decreased in mutants. B. Immunostaining of Sall1. Sall1 protein expression is also decreased in mutants. Lower: higher magnification of areas shown in the white rectangles. C. Active β-Catenin is determined by immunoblotting with a β-Catenin antibody that detects unphosphorylated residues at Ser33/Ser37/Thr41, which target the molecule for ubiquitination. β-Catenin is activated in mutants. D. RT-PCR of *Wnt9b* and *Axin2*. The mRNA levels of *Wnt9b* and *Axin2*, a target gene of Wnt/β-Catenin signaling, are increased in mutants. E. semi-quantitative RT-PCR for *Kif26b*. The mRNA level of *Kif26b*, a target gene for Sall1, is reduced in mutants. nor—normal, mu—mutant.

Branching morphogenesis is regulated by reciprocal interactions between the MM and UB [2]. Since *Ifng* expression was targeted to the MM, we first examined the expression of regulatory molecules that are involved in kidney morphogenesis and that are also localized to the MM. For this, we examined the expression of Gdnf, a key factor in kidney branching morphogenesis, and Gdnf-regulating molecules, Eya1, Six1, and Pax2 [43,44]. *Gdnf* expression in the kidney at E10.5 was normal (S3A Fig) as were the expressions of *Eya1*, *Six1* and *Pax2* by semi-quantitative RT-PCR (S3B Fig). Sall1 is also involved at the initiation of kidney development by regulating UB invasion and primary branching [3–5] and by maintaining the multipotency of renal progenitors [6]. It is normally localized to the MM and affects the survival of renal progenitors during early kidney development [3]. The *Ifng* GOF mutant kidneys do exhibit a phenotype reminiscent of the kidneys from the *Sall1* loss-of-function mouse, which also shows renal agenesis or dysplasia [3,5]. Furthermore, *Sall1* is expressed in renal stromal progenitors, which overlay the Six2+/Pax2+ MM cells [45] and in which we observe an increase in cell death (Fig 3D). Finally, Ifng expression in mutants is especially increased (S2A Fig) where Sall1 is normally expressed [46]. In the current study, *Sall1* was decreased in GOF mutant kidneys as measured by semi-quantitative RT-PCR (Fig 5A). By confocal microscopy, Sall1 protein levels were reduced, especially in the renal cortex (Fig 5B) as well as in the MM during the initiation of nephrogenesis at E10.5 (S4A Fig).

Sall1 activity in the MM nonautonomously modulates canonical Wnt9b signaling in the UB tip to facilitate branching [5]. Therefore, we examined canonical Wnt activity by probing kidney lysates for active β-Catenin in the *Ifng* GOF mutants. To detect the active form, we used a commercial antibody raised against a β-Catenin epitope (aa36-44) which is dephosphorylated at Ser33/Ser37/Thr41 and thus stabilized from proteasomal degradation by post-translational ubiquitination [47]. The level of active β-Catenin protein increased in the *Ifng* GOF mutant kidneys (Fig 5C). Furthermore, the expression of UB-localized *Wnt9b*, which regulates branching morphogenesis through canonical Wnt/β-Catenin signaling, was increased in the *Ifng* GOF mutants (Fig 5D) and ectopically localized in the UB stalk (S4B Fig), as reported for the *Sall1* loss-of-function mouse [5]. *Axin2*, a target gene of canonical Wnt/β-Catenin signaling, was also increased in mutants (Fig 5D). Additionally, the expression of *Kif26b*, a downstream target of Sall1 which facilitates UB/MM cell adhesion [4], was decreased in the *Ifng* GOF mutant kidneys, indicating that the reduction of Sall1 may also limit expression of *Kif26b* and thus inhibit the adhesion of MM cells to the UB (Fig 5E). Taken together, these results suggest that an MM-specific increase in Ifng may cause renal agenesis or hypoplasia by regulating Sall1 expression, which is critical for the survival of renal progenitors, UB invasion, [3,4] branching morphogenesis, [5] and maintenance of multipotent renal progenitors [6]. Finally, these results raise the possibility that progenitors in other tissues may lose their multipotency as a result of transplacental induction of Ifng overexpression and thus cause aberrant development as suggested by studies of the brains or eyes of Zika-infected fetuses [48].

## Discussion

In this study, we analyzed the effect of MM-specific Ifng expression during kidney development using an *Ifng* GOF mouse. The metanephric rudiment in mutant embryos initially formed as expected, but as the UB began arborizing in the MM, its progression was clearly retarded, yielding minimally a hypoplastic kidney. Moreover, cell death was prominent in mutants in and around the MM during early development, and this likely accounts for the observed unilateral or bilateral renal agenesis. All these phenotypes are similar to those described for the *Sall1* mutant mouse [3,5]. We confirmed the decreased expression of *Sall1* mRNA and protein in the *Ifng* GOF mutant mice, although levels reflected a general reduction in Sall1 rather than its complete loss as in the Sall1 knockout kidney. Regardless, the mutant kidneys showed an ectopic expression of *Wnt9b* and thus increased expression of the active form of β-Catenin as well as Wnt/β-Catenin target gene *Axin2*, as were demonstrated by others for the *Sall1* mutant [5]. Conversely, the expression of Sall1 downstream target gene *Kif26b* [4] was decreased in the *Ifng* mutant kidneys, as was also observed in the *Sall1* KO mouse. These data are consistent with the possibility that the kidney phenotype in the *Ifng* GOF mouse is the result of decreased expression of Sall1 in the mutant kidneys.

*Sall1* knockout kidneys are characterized by the decreased expression of several progenitor stemness genes, including *Osr1*, *Eya1*, *Pax2*, *Cited1*, and *Six2* by RT-qPCR [46,49]. While we observed no apparent reductions in the expression of Pax2 or Eya1 by semi-quantitative RT-PCR of our tissues, the differentials reported for the Sall1 KO kidneys were significant but relatively small. The absence of a differential for our mutant kidneys, in which there is only a partial reduction in Sall1 expression, is therefore not surprising. Where this may be relevant is in the cells of the Ifng GOF mutant kidneys that die. We noted earlier that the Tunel+ cells in sections from E11.5 kidneys apparently lack co-expression of *Pax2*. If these cells in particular were among those with reduced Sall1 expression, which presumably caused their death, then it is reasonable to assume that the Sall1 deficiency may have also precipitated the decrease or loss of Pax2 within that population.

In our previous study, we reported that Ifng treatment of isolated MM induces the proliferation of MM cells and inhibits their differentiation, i.e., mesenchymal-epithelial transition (MET) [34]. From these results, we speculated that the expression of *Ifng* in the MM might induce uncontrolled proliferation of MM cells, which provide the putative stem cells for Wilms tumors/nephroblastoma [50,51]. This prediction was also based upon reports that ectopic expression of *Ifng* in astrocytes during early postnatal development induces proliferation in neural progenitors and a high frequency of medulloblastomas [25]. If *Ifng* levels were sustained in these animals, the developing tumors were replete with apoptotic cells. Our model induces sustained expression of Ifng in the MM area, but we did not observe an increase in MM proliferation *in situ* with ectopic expression of Ifng or evidence of nephroblastoma formation. We did, however, detect increased cell death during early kidney development, as reported in the neural studies. Thus, the phenotype of constitutive expression of *Ifng* in the MM is not proliferation, but rather cell death during early kidney development and retarded UB branching through the putative regulation of Sall1-positive renal progenitors. Is this relevant though to instances of activation of innate immunity following transplacental infection? One might argue that constitutive ectopic expression of Ifng, as occurs in the transgenic mice, does not reflect the typical kinetics associated with transplacental viral transduction and Ifng induction. In the limited studies in which these kinetics have been examined, Ifng levels peaked at 72-hrs post infection and remained significantly elevated after 96 hrs in embryos [52]. Should infection occur during the critical early stages of metanephric development, e.g. E10.5-E11.5 in mice, a 72–96 hr window of exposure to Ifng could reasonably impact branching morphogenesis thereby reducing nephron numbers. However, a direct assessment of this speculative outcome remains to be performed.

Sall1 expression is essential for kidney development and regeneration by regulating the survival and multipotency of the renal progenitors [3–6]. Since our data suggest that Ifng modulates Sall1 expression, it is possible that transplacental viral infections may induce Ifng expression, [48] which, in turn, might eliminate progenitors or disrupt their multipotency by regulating genes which are responsible for maintenance of stemness, as is the case for Sall1 in renal development. While speculative, it is also conceivable that the abnormal development of the eye or brain in children infected transplacentally by Zika virus, for example, [48] may be caused in part by increased Ifng, as observed in Ifng overexpression mouse models [25,31].

Besides genetic defects, extrinsic factors can affect kidney development [1,19]. As reported for embryonic neural tissues [26], *Ifng* receptors are expressed at high levels both in the MM and UB of the embryonic kidney (S1 Fig). Also, we could detect very weak expression of *Ifng* mRNA normally in isolated MM (S1 Fig). During development, macrophages, a principal source for Ifng, are formed in the yolk sac and migrate into most tissues of the mouse embryo by E10.5. They have been detected in the developing urogenital tract by the time nephrogenesis is initiated [53] and are therefore a potential intrarenal source of Ifng with transplacental infection. The placenta itself may also bathe the developing embryo with the cytokine during active infections; however, such responses have been linked to spontaneous abortions [54,55].

A 60-fold induction of *Ifng* transcript levels has been reported in embryos following in utero viral inoculation, [52] and these levels may reasonably reflect the 13-fold increase in protein observed in our rudiments on the basis of studies involving comparative assessments of multiple RNA/protein levels [56]. In our model, levels were determined in mutant hypoplastic kidneys and, therefore, may not reflect levels achieved in the instances of the more profound phenotype of renal agenesis. Moreover, the receptor expression patterns suggest that transplacental infection with pathogens that elicit an innate immune response at certain stages of development may impact the proper patterning of organs such as the kidney through elevated Ifng expression. Indeed, MM-targeted expression of *Ifng* caused a retardation in kidney development. We also tested the expression of *Ifng* in the UB using an AP2Cre mouse, which induces gene recombination in the UB but not MM cells [57]. In this case, the expression of *Ifng* did not yield any kidney phenotype (personal observation). While this may indicate that Ifng signaling affects only the MM progenitor cells, increased expression of IRF6 in the UB suggests that the secreted ligand from the MM also signals to the UB epithelia. More likely, the absence of a phenotype in the AP2Cre kidney may be due to the more limited activation of Ifng expression with this Cre line.

Regarding a role for Ifng in development, it is known that the administration of this cytokine induces not only fetal abortion [58], but also retardation of organ development as reported for the eye [31]. An *Ifng* transgenic mouse yielded abnormalities of the eye [31] or brain involving an inhibition of differentiation and tissue dysplasia [25]. These findings suggest that the induction of innate immunity in the form of Ifng, rather than a transplacental pathogen may be responsible for some sequelae associated with infection during pregnancy. In the kidney, even small reductions in branching morphogenesis could result in long-term health consequences, such as hypertension and hyperfiltration [21]. Thus, the *Ifng* GOF mouse may provide an important model for delineating the role of innate immune responses in aberrant tissue morphogenesis during embryonic development.

## Supporting information

S1 Fig *Ifng* is marginally expressed in the MM (40 PCR cycles) of E11.5 kidneys, while its cognate receptors are readily detected in both the MM and UB (33 PCR cycles). RNA from MM and UB were prepared from normal kidneys at E11.5 after tissue separations by trypsinization. *Six2* and *Gdnf* are markers of MM, and *c-Ret* is a marker for the UB. PCR conditions, including cycle numbers are shown in S1 Table.
(TIF)

S2 Fig Expression of IRF6 is increased in nephronic and UB epithelia in E14.5 kidneys. Lower: higher magnification of area delineated in the white rectangle. The UB is identified by a Hoxb7/myr-venus reporter.
(TIF)

S3 Fig The expression of genes involved in early kidney development does not change in *Ifng* GOF mutant kidneys. A. WISH for *Gdnf* in embryos at E10.5. B. expression of *Eya1*, *Six1*, *Pax2*, and *Gdnf* in kidneys at E14.5 by semi-quantitative RT-PCR.
(TIF)

S4 Fig Aberrant expression of Sall1 and *Wnt9b* in mutant kidneys. A. The expression of Sall1 is diminished in mutant kidneys at E10.5. The white dotted circle delineates the metanephros prior to UB invasion. B. WISH of *Wnt9b* at E11 (normal) or at E11.5 (mutant). *Wnt9b* is ectopically expressed in the UB stalk of the mutant (arrowhead). nor—normal, mu—mutant, ND—nephric duct, CND—common nephric duct.
(TIF)

S5 Fig Original blots from which data included in the publication were derived. (PDF)

S1 Table Primers used in semi-quantitative RT-PCR analyses. (DOCX)

## Abbreviations

CAKUT: congenital anomalies of the kidney and urinary tract
GOF: gain-of-function
Ifng: Interferon-gamma
KO: knockout
MM: metanephric mesenchyme
UB: ureteric buds
WISH: whole-mount in situ hybridization
