# Supplementary figures and images for: Constitutive metanephric mesenchyme-specific expression of interferon-gamma causes renal dysplasia by regulating Sall1 expression
Source: PLoS One. 2018 May 17;13(5):e0197356. doi: 10.1371/journal.pone.0197356 (PMC5957351; doi:10.1371/journal.pone.0197356)

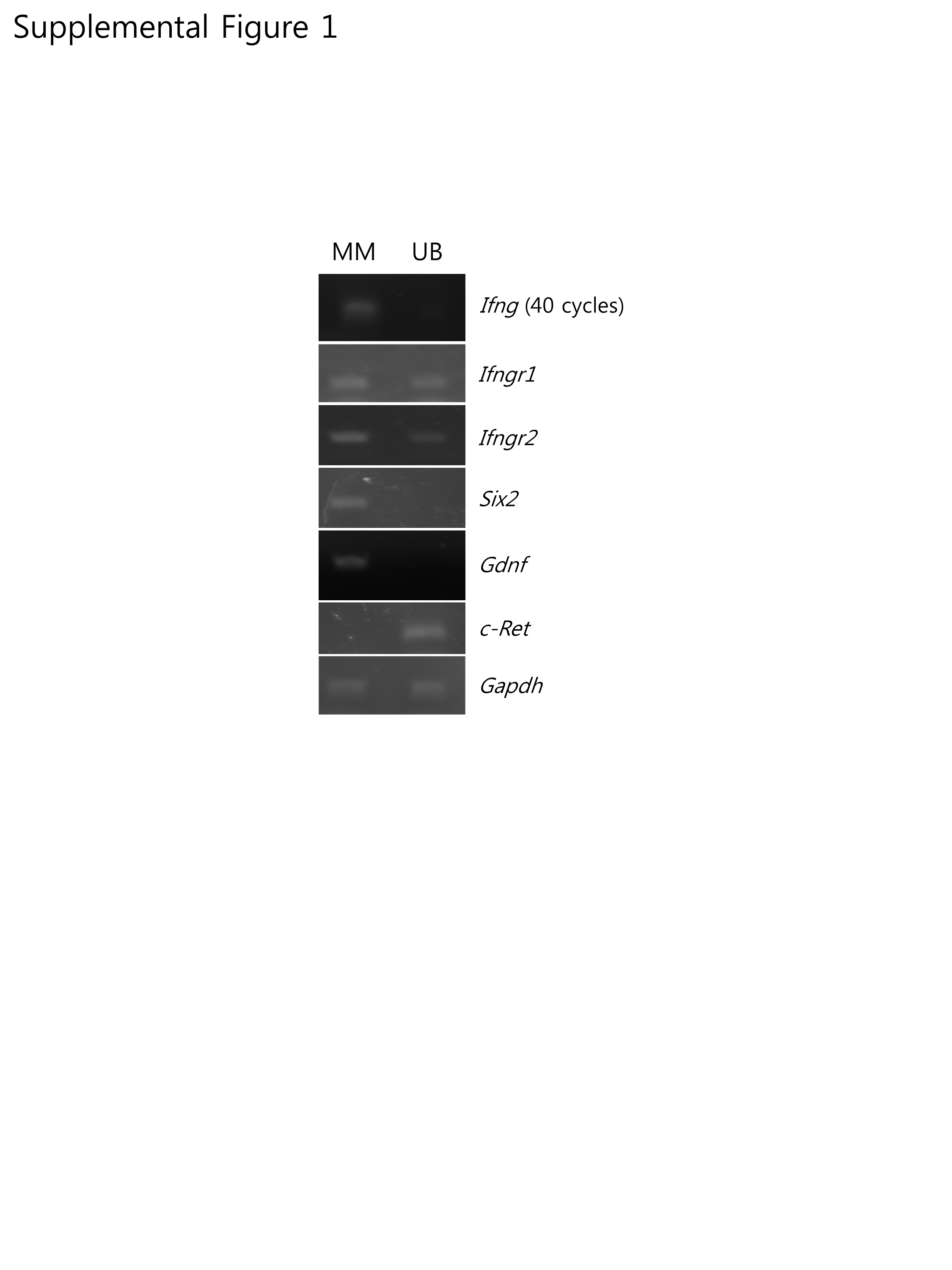

Supplement: S1 Fig — RNA from MM and UB were prepared from normal kidneys at E11.5 after tissue separations by trypsinization. Six2 and Gdnf are markers of MM, and c-Ret is a marker for the UB. PCR conditions, including cycle numbers are shown in S1 Table. (TIF) [file pone.0197356.s001.TIF]

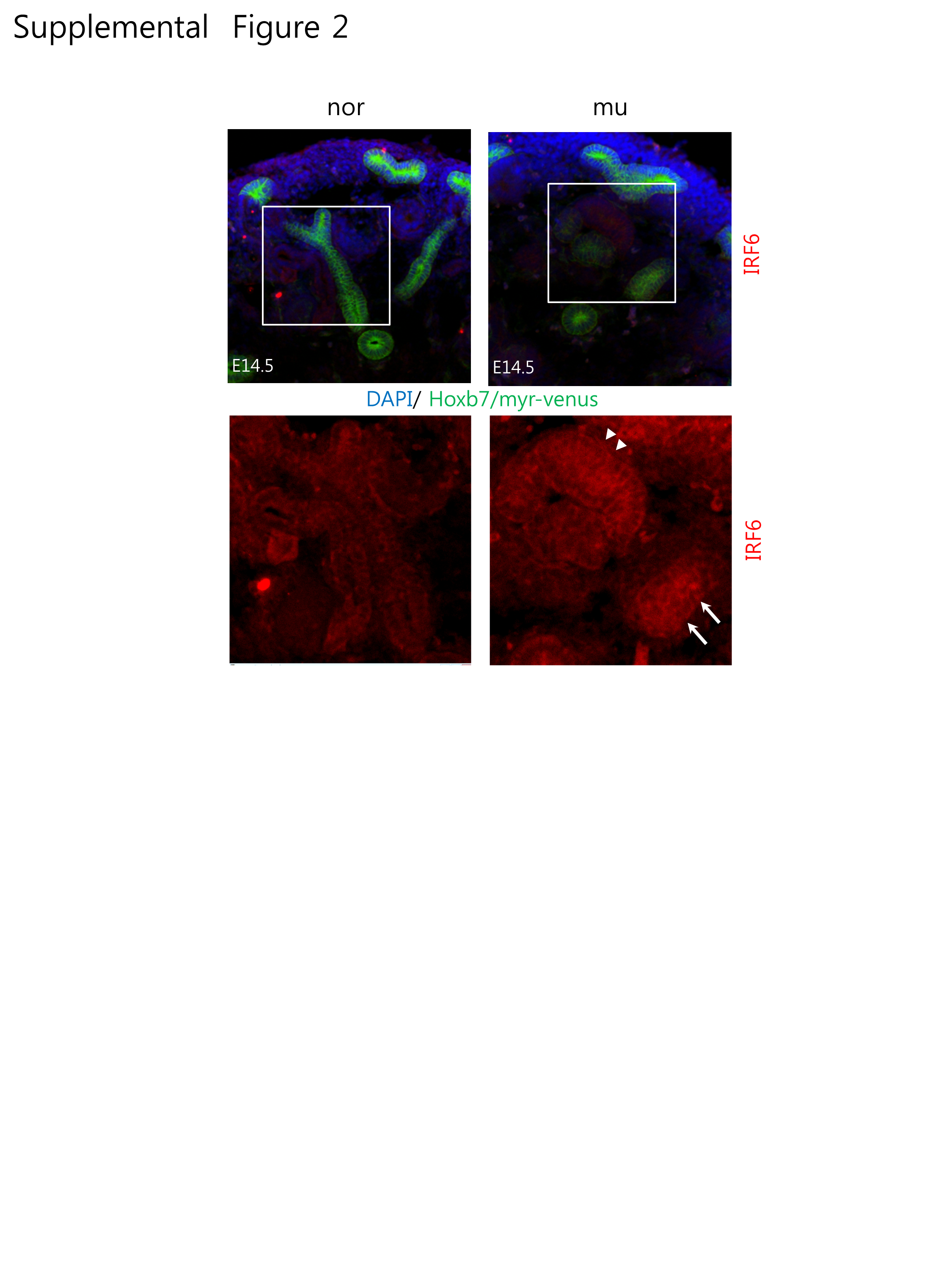

Supplement: S2 Fig — Lower: higher magnification of area delineated in the white rectangle. The UB is identified by a Hoxb7/myr-venus reporter. (TIF) [file pone.0197356.s002.tif]

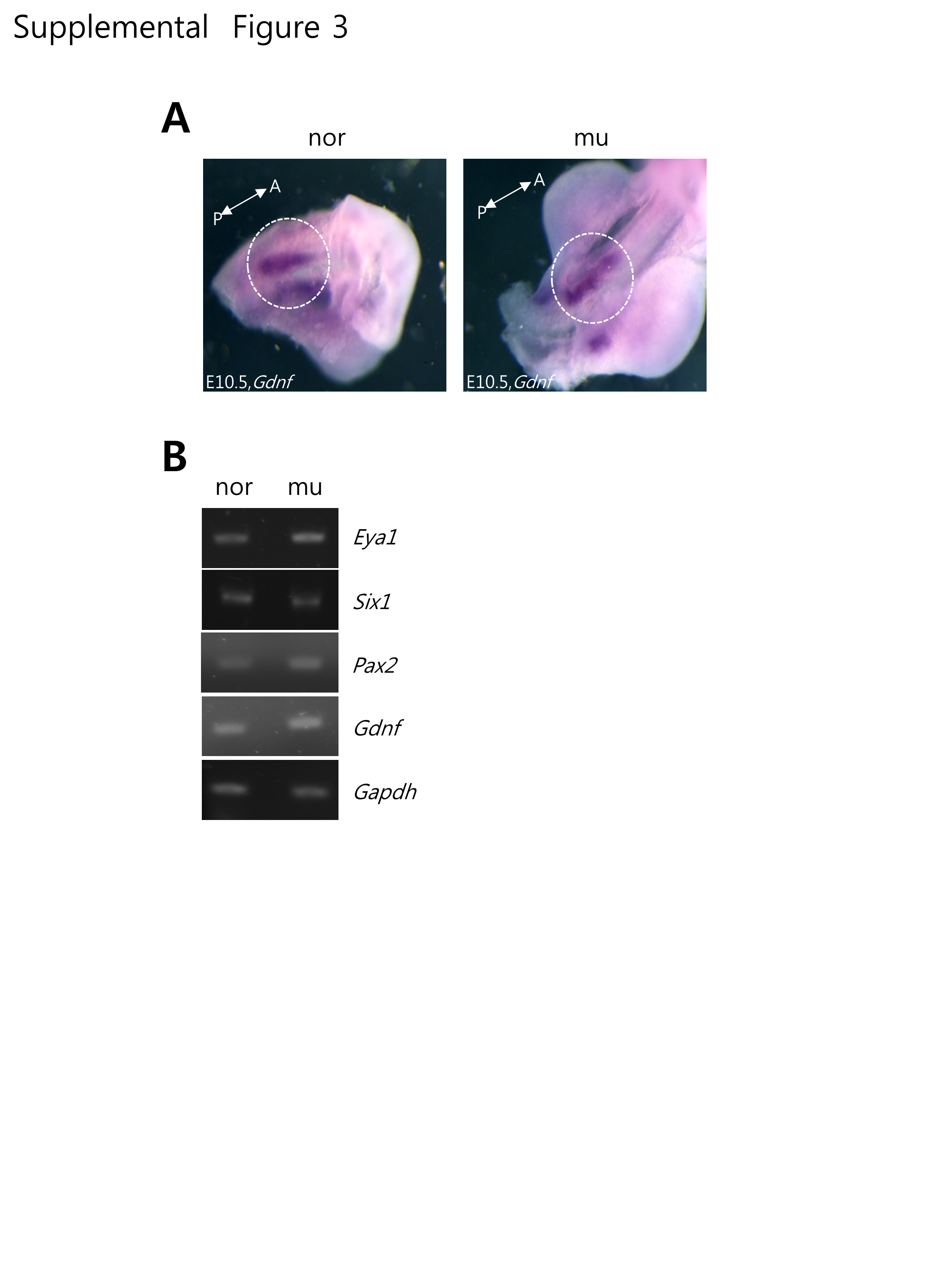

Supplement: S3 Fig — A. WISH for Gdnf in embryos at E10.5. B. expression of Eya1, Six1, Pax2, and Gdnf in kidneys at E14.5 by semi-quantitative RT-PCR. (TIF) [file pone.0197356.s003.TIF]

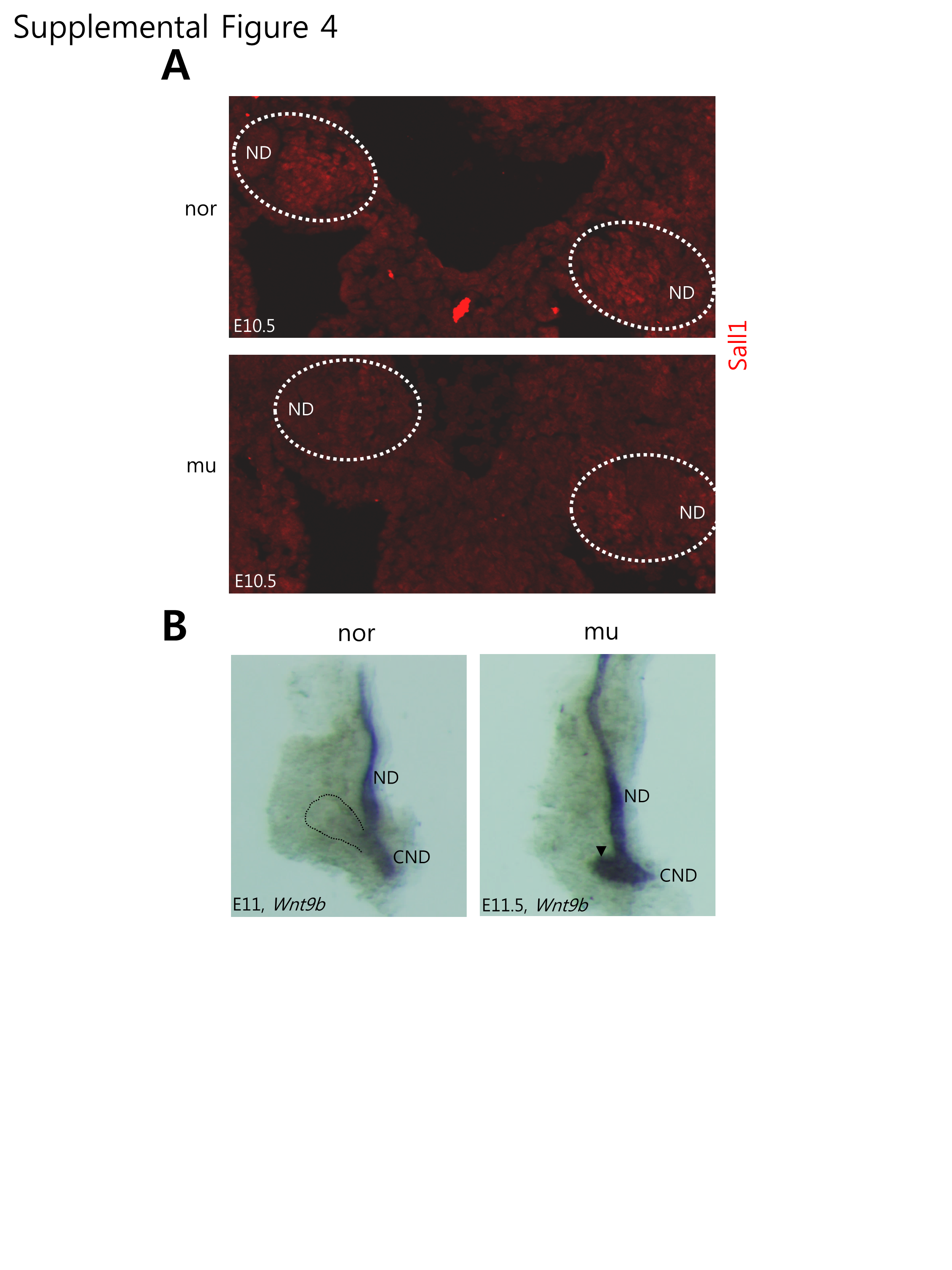

Supplement: S4 Fig — A. The expression of Sall1 is diminished in mutant kidneys at E10.5. The white dotted circle delineates the metanephros prior to UB invasion. B. WISH of Wnt9b at E11 (normal) or at E11.5 (mutant). Wnt9b is ectopically expressed in the UB stalk of the mutant (arrowhead). nor—normal, mu—mutant, ND—nephric duct, CND—common nephric duct. (TIF) [file pone.0197356.s004.tif]
