# Supplementary material for: Constitutive metanephric mesenchyme-specific expression of interferon-gamma causes renal dysplasia by regulating Sall1 expression
Source: PLoS One. 2018 May 17;13(5):e0197356. doi: 10.1371/journal.pone.0197356 (PMC5957351; doi:10.1371/journal.pone.0197356)
Supplement: S5 Fig — (PDF) [file pone.0197356.s005.pdf]

Figure 1B & Figure 5D

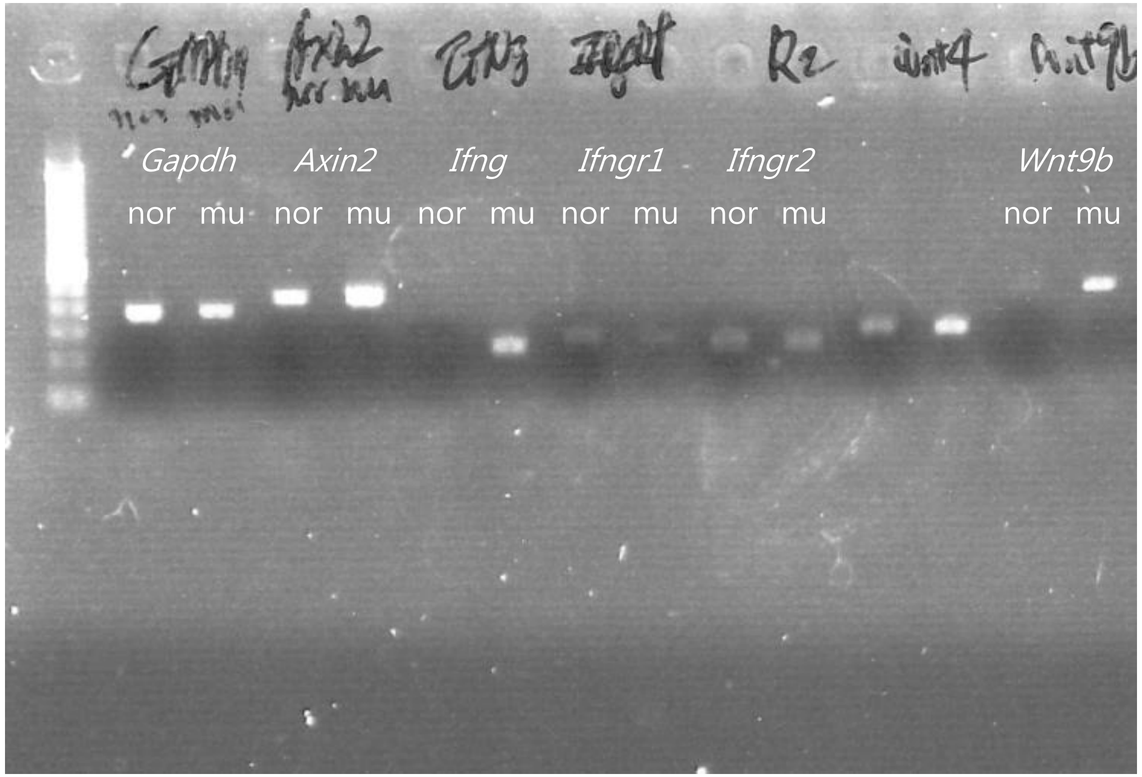

Lost data saved in computer→ scanned from notebook

Figure 1C

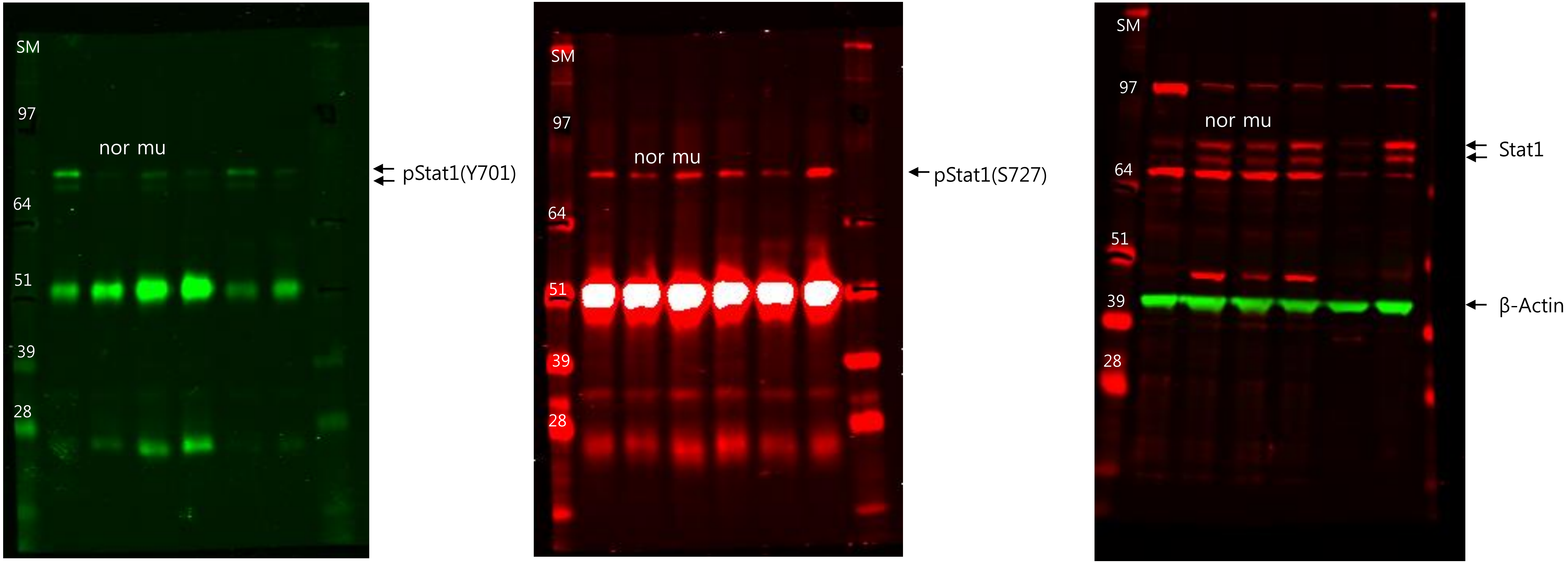
SM
97
nor mu
64
51
39
28
pStat1(Y701)
SM
97
nor mu
64
51
39
28
pStat1(S727)
SM
97
nor mu
64
51
39
28
Stat1
β-Actin

Figure 5A & Supplemental Figure 3

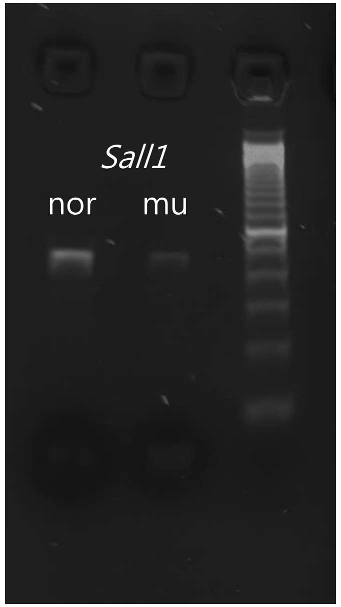

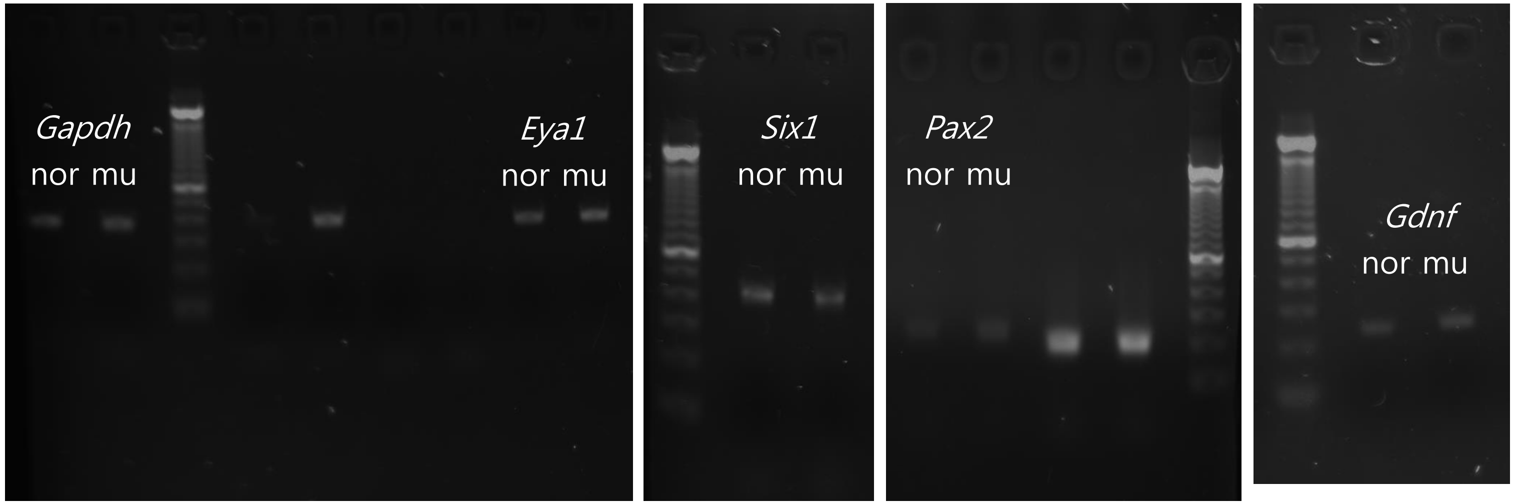

Figure 5C

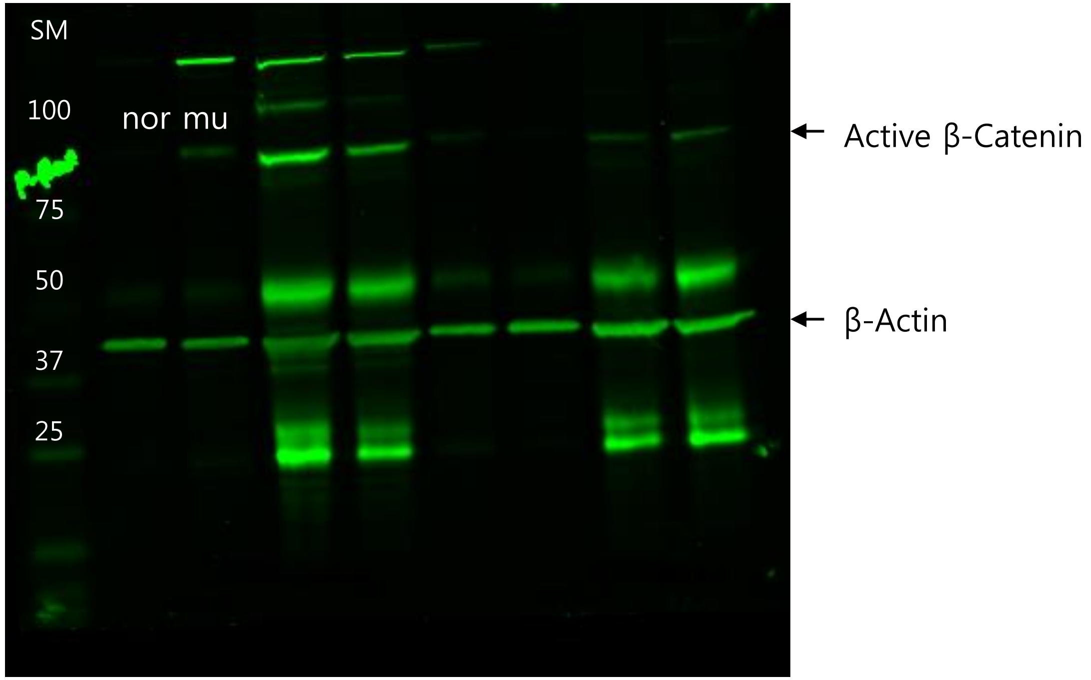

Figure 5E

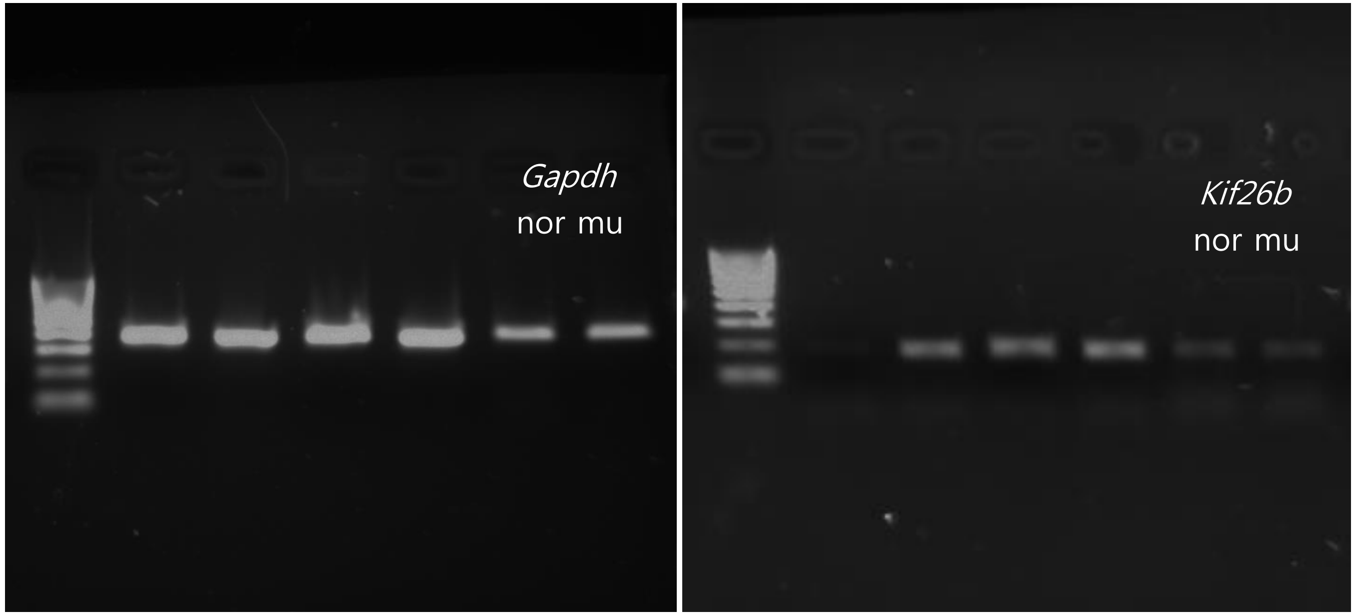

Supplemental Figure 1

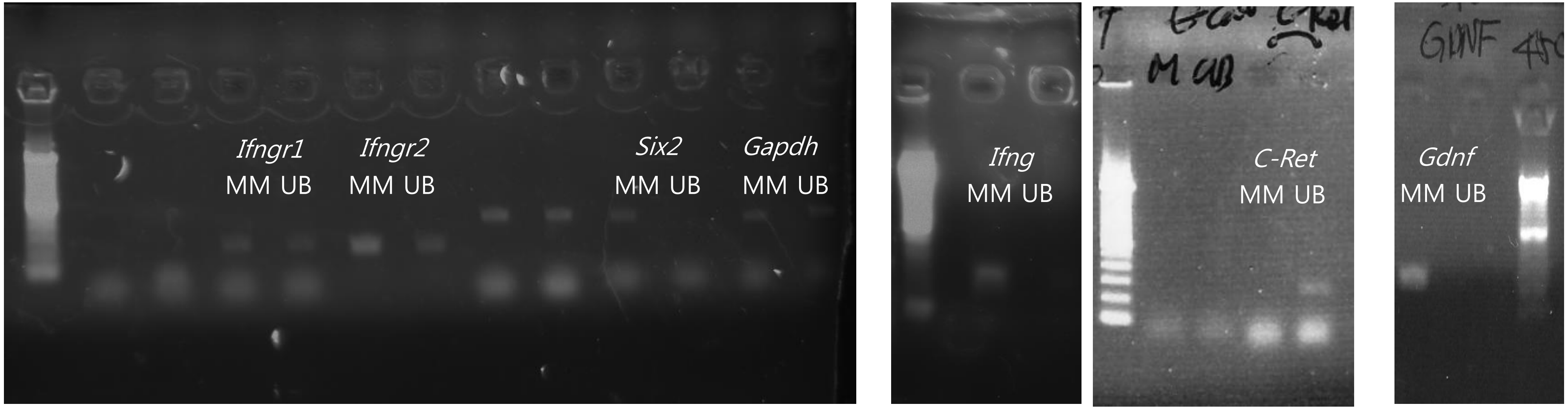

Lost data saved in computer→
scanned from notebook
