# Supplementary material for: Constitutive metanephric mesenchyme-specific expression of interferon-gamma causes renal dysplasia by regulating Sall1 expression
Source: PLoS One. 2018 May 17;13(5):e0197356. doi: 10.1371/journal.pone.0197356 (PMC5957351; doi:10.1371/journal.pone.0197356)
Supplement: S1 Table — (DOCX) [file pone.0197356.s006.docx]

### S1 Table: Primers used in semi-quantitative RT-PCR analyses.

| Gene | Sequence (5'-3') | Product size(bp) | Annealing temparature (°C) | Cycle |
| --- | --- | --- | --- | --- |
| *Axin2* | F cattttggacgaccacctct | 424 | 60 | 37 |
|  | R ttttggcaaggtaccacctc |  |  |  |
| *Eya1* | F gacctggacgagaccatcat | 384 | 60 | 33 |
|  | R cagacctcccacgttgtttt |  |  |  |
| *Gdnf* | F ccccgaagccatctgttccaaa | 242 | 60 | 35 |
|  | R tagcccaaacccaagtcagt |  |  |  |
| *Gapdh* | F cccttcattgacctcaactacatgg | 368 | 60 | 27 |
|  | R gstaagcagttggtggtgcagg |  |  |  |
| *Ifng* | F actggcaaaaggatggtgac | 212 | 60 | 37/40 |
|  | R gacctgtgggttgttgacct |  |  |  |
| *Ifngr1* | F gggttcctggactgattcct | 229 | 60 | 33 |
|  | R tacgaggacggagagctgtt |  |  |  |
| *Ifngr2* | F gcttcaccctgttcctcaaa | 205 | 60 | 33 |
|  | R agcacatcatctcgctcctt |  |  |  |
| *Pax2* | 5'cccacattagaggaggtgga3' 212 | | 60 | 35 |
|  | 5'gatgtgctctgatgcttgga3' |  |  |  |
| *Sall1* | F atccagatgaacccctaccc | 466 | 60 | 37 |
|  | R acagagggttggtgaaggtg |  |  |  |
| *Six1* | F ctggagagccaccagttctc | 392 | 60 | 40 |
|  | R tgcttgttggaggaggagtt |  |  |  |
| *Six2* | F caagaatgaaagcgtgctca | 386 | 60 | 33 |
|  | R tgaaccagttgctgacttgc |  |  |  |
| *Wnt9b* | F cctgctccagagaggcttta | 418 | 60 | 37 |
|  | R gacagccgtgtcatagcgta |  |  |  |
